# Changes in Expression in BMP2 and Two Closely Related Genes in Guinea Pig Retinal Pigment Epithelium during Induction and Recovery from Myopia

**DOI:** 10.3390/biom13091373

**Published:** 2023-09-11

**Authors:** So Goto, Yan Zhang, Sonal Aswin Vyas, Qiurong Zhu, Christine F. Wildsoet

**Affiliations:** 1 Herbert Wertheim School Optometry and Vision Science, University of California, Berkeley, CA 94720, USA; 2 Department of Ophthalmology, Osaka University Graduate School of Medicine, Suita 565-0871, Japan; 3 Department of Ophthalmology, National Hospital Organization, Tokyo Medical Center, Meguro-ku, Tokyo 152-8902, Japan; 4 Schepens Eye Research Institute, Massachusetts Eye and Ear, Harvard Medical School, Boston, MA 02114, USA

**Keywords:** myopia, recovery, gene expression, retinal pigment epithelium, bone morphogenetic protein 2 (Bmp2)

## Abstract

Purpose: We previously reported differential gene expression of the bone morphogenetic protein 2 (*Bmp2*) in guinea pig retinal pigment epithelium (RPE) after 1 day of hyperopic defocus, imposed with a negative contact lens (CLs). The study reported here sought to obtain insights into the temporal profiles of gene expression changes in Bmp2, as well as those of two closely related genes, the inhibitor of DNA binding 3 (Id3) and Noggin (Nog), both during myopia induction and when the CL treatment was terminated to allow recovery from induced myopia. Methods: To induce myopia, 2-week-old pigmented guinea pigs (New Zealand strain, n = 8) wore monocular −10 diopter (D) rigid gas-permeable (RGP) CLs for one week, while the other eye served as a control. Ocular measurements were made at baseline, 3 days, and 7 days after the initiation of CL wear, with treatment then being terminated and additional measurements being made after a further 3 days, 1 week, and 2 weeks. Spherical equivalent refractive errors (SERs), axial length (AL), choroidal thickness (ChT), and scleral thickness (ScT) data were collected using retinoscopy, optical biometry (Lenstar), and spectral domain optical coherence tomography (SD-OCT), respectively. RPE samples were collected from both eyes of the guinea pigs after either 1 day or 1 week of CL wear or 1 day or 2 weeks after its termination, and RNA was subsequently isolated and subjected to quantitative real-time PCR (qRT-PCR) analyses, targeting the *Bmp2*, *Id3*, and *Nog* genes. Results: Mean interocular differences (treated—control) in AL and SER were significantly different from baseline after 3 and 7 days of CL wear, consistent with induced myopia (*p* < 0.001 for all cases). Termination of CL wear resulted in the normalization (i.e., recovery) of the ALs and SERs of the treated eyes within 7 days, and the earlier significant ChT thinning with CL wear (*p* = 0004, day 7) was replaced by rapid thickening, which remained significant on day 7 *(p* = 0.009) but had normalized by day 14. The ChT changes were much smaller in magnitude than the AL changes in both phases. Interocular differences in the ScT showed no significant changes. The Bmp2 and Id3 genes were both significantly downregulated with CL wear, after 1 day (*p* = 0.012 and 0.016) and 7 days (*p* = 0.002 and 0.005), while Bmp2 gene expression increased and Nog gene expression decreased after the termination of CL wear, albeit transiently, which was significant on 1 day (*p* = 0.004 and 0.04) but not 2 weeks later. No change in Id3 gene expression was observed over the latter period. **Conclusions:** The above patterns of myopia induction and recovery validate this negative RGP-CL model as an alternative to traditional spectacle lens models for guinea pigs. The defocus-driven, sign-dependent changes in the expression of the Bmp2 gene in guinea pig RPE are consistent with observations in chicks and demonstrate the important role of BMP2 in eye growth regulation.

## 1. Introduction

Myopia (near-sightedness) is most commonly caused by excessive ocular axial elongation [1,2]. Due to the more recent dramatic increases in the prevalence of myopia worldwide [1,2], myopia has also become a significant public health problem socially and economically [3]. As myopia progresses, the risks of serious ocular complications such as retinal detachment, glaucoma, and cataracts increase exponentially, with the first two potentially leading to permanent vision loss [4]. Much has been learned through research about the underlying pathophysiological changes in and key risk factors for developing high myopia, both of which have been well characterized, the former including thinning of the choroid and sclera, along with increased axial length and possible posterior staphylomas [5,6,7], and the latter including the early onset of myopia. However, the molecular and cellular mechanisms driving the early accelerated eye elongation that underlies myopia development and progression remain poorly understood. This limited understanding constrains the development and scope of effective therapies for controlling myopia.

Research using animal models for myopia suggests that early developmental eye growth, leading to refractive errors, operates under local ocular control and is visually regulated. The process appears to originate from signals in the retina, which are then relayed through the retinal pigment epithelium (RPE) to the vascular choroid and finally to the sclera [8,9]. These two layers together determine the state of focus of the eye, i.e., its refractive error, through their direct or indirect influences on the position of the retina relative to the eye’s optical components and thus the focal plane of the eye. The RPE has a significant role in early choroidal development and postnatally, functional support of the outer retina [10,11]. Based on its critical location, interposed between the retina and choroid, it may play a role as a signal relay in eye growth regulation and thus in the development of myopia [8,12]. With respect to molecular mechanisms, the outer choroid and sclera have been the focus of most research in the context of myopia development to date [13,14,15,16], with the roles of dopamine, as a key neurotransmitter, and neuropsin, as a key photopigment, receiving enduring attention in studies involving the retina, especially in recent years [17,18,19].

In the context of eye growth regulation and myopia, relatively little attention has been directed toward understanding the role of the RPE, likely in part due to the technical challenges associated with its study. Much of the relevant research into the role of RPE as a potential source of growth modulators is summarized in a 2020 review, which shows heavy reliance on the chick as an animal model for such studies [20]. A number of multifunctional growth factors have thus been implicated, including members of the transforming growth factor beta (TGF β) super family, basic fibroblast growth factor (bFGF), and vascular endothelial growth factors (VEGFs). Members of the former group include bone morphogenetic proteins (BMPs), which are now recognized as playing important roles in eye organogenesis, morphogenesis, and early development across a variety of animal models [21,22]. Specific to induced myopia-related changes in eye growth, we recently reported downregulation in BMP gene expression tied to the early phase of myopia induction in guinea pigs [23]. This finding parallels similar trends observed in the RPE of chicks exposed to myopia-inducing conditions, such as negative optical defocus and form deprivation [24,25].

The purpose of the study described here was to further investigate the changes in *Bmp2* gene expression in the RPE of guinea pigs with imposed hyperopic defocus, hereafter referred to as lens-induced myopia (LIM), and to also investigate gene expression changes under the opposing, myopic defocus condition, as experienced after the termination of myopia-inducing lens wear. At least in young animals, rapid recovery from induced myopia is the expected result. Changes in the RPE of the expression of two additional genes, the inhibitor of DNA binding 3 (*Id3*) gene, which is a target of BMP, and Noggin (*Nog*), an antagonist of BMP, were also investigated in parallel. We used monocular negative power contact lenses (CLs) to induce myopia and studied changes in both ocular dimensions and RPE gene expression at timed intervals, during the induction period, as well as after the lens treatment was terminated. As previously observed in related studies in chicks, we observed bidirectional changes in *Bmp2* expression, in line with the imposed sign and magnitude of optical defocus, downregulation with imposed hyperopic defocus and upregulation with induced myopia defocus, with changes in the expression of *Id3* and *Nog* genes that could be only partly predicted by the known relationship between these three genes.

## 2. Methods

### 2.1. Animals

Two-week-old pigmented guinea pigs were used in this study, with breeders obtained from the University of Auckland (Auckland, New Zealand). The pups were bred on-site and weaned at 7 days of age. For this study, they were reared as single-sex pairs in transparent plastic tubs (41 × 51 × 22 cm) in a temperature-controlled room. Room lighting was set to a 12 h light/12 h dark cycle (on at 9:45 a.m.; off at 9:45 p.m.), with an average cage floor luminance of 160 to 180 lux. The animals had free access to water and were fed a high-fiber guinea pig diet (Teklad 2041, Envigo, Madison, WI, USA), along with fresh fruit and vegetables three times a week as dietary enrichment. All animal care and treatments used in this study conformed to the ARVO Statement for the Use of Animals in Ophthalmic and Vision Research. The experimental protocols were also approved by the Animal Care and Use Committee of the University of California, Berkeley.

### 2.2. Rigid Gas-Permeable Contact Lense-Induced Myopia Model

Myopia was induced using a −10 diopter (D) rigid gas permeable (RGP) contact lens (CL), inserted in the right eye only. Details concerning the lens design, as well as lens wear and monitoring schedules, are described in a previous publication [26]. Briefly, the CLs (Valley Contax, Springfield, OR, USA) were made from acrylic fluorosilicone material, which has high oxygen permeability (65%), and custom designed for our guinea pig subjects (overall diameter: 6.00 mm, optic zone diameter: 5.00 mm, and base curve: 3.38 mm). A continuous wear schedule was initiated at 14 days of age and continued for up to 7 days, with lenses removed and replaced with clean lenses every morning and otherwise checked three times a day. In between use, the lenses were soaked in a combination of Boston protein remover and Boston Simplus solution (Bausch and Lomb, Rochester, NY, USA), with thorough rinsing with Opti-Free soft contact lens solution (Alcon, Fort Worth, TX, USA) prior to insertion. Figure S1 summarizes the key features of the study design, including the duration of CL wear (myopia induction) and following the recovery period, i.e., after discontinuation of CL wear, along with the timing of in vivo biometric measurements and the collection of retinal pigment epithelium (RPE) samples.

### 2.3. Biometric Data Collection

#### 2.3.1. Refractive Errors and Axial Lengths

Refractive error and axial length (AL) data were collected from awake animals at baseline, as well as after 3 and 7 days of CL wear, when lens wear was then discontinued and additional measurements were taken 3, 7, and 14 days later. Measurements on individual animals were performed at the same time of day, at approximately 2:00 p.m., to avoid possible confounding effects of circadian rhythms on eye growth.

Refractive errors were measured by two researchers (SG and QZ) using streak retinoscopy (Welch Allyn, Skaneateles Falls, NY, USA) following cycloplegia with 1% cyclopentolate hydrochloride (Bausch & Lomb, Rochester, NY, USA), instilled 30 min prior to measurement. The results are reported as spherical equivalent refractive errors (SERs; average of results for the two principal meridians). An optical biometer (Lenstar; Haag-Streit Holdings, Köniz, Switzerland) was used to measure axial lengths (ALs), which, as reported here, refer to the distance from the anterior surface of the cornea to the inner surface of the retina. Each measurement comprised an average of at least five readings.

#### 2.3.2. Choroidal and Scleral Thicknesses (SD-OCT)

Spectral-domain optical coherence tomography (SD-OCT, Envisu R-2300; Bioptigen, Morrisville, NC, USA) was used to obtain choroidal thickness (ChT) and scleral thickness (ScT) data. For these measurements, the guinea pigs were anesthetized with a ketamine/xylazine cocktail (27/0.6 mg/kg body weight) and positioned on a customized platform for imaging. The SD-OCT scanning protocol used in this study was as previously described, i.e., 70 B-scans and 700 A-scans, with 30 frames per B-scan and a 2.6 × 2.6 mm-wide field of view. Analyses were restricted to the visual streak region, which is approximately 1000 μm away from the center of the optic nerve head (ONH), with the latter being used as a reference landmark in comparing images captured from the same animal at different timepoints over the study period. The middle third of the captured cross-sectional images was selected for analysis to avoid optical distortions affecting the more peripheral parts of the images, with the built-in calipers used to measure ChT and ScT. ChT was measured as the perpendicular distance between the outer boundary of the retinal pigment epithelium (hyperreflective line) and the choroid–sclera interface, while ScT was measured as the perpendicular distance between the choroid–sclera interface and outer scleral boundary. The results represent the averages of measurements at 3 points, i.e., at the center and 500 um away on each side of the center of the image.

### 2.4. Retinal Pigment Epithelium (RPE) Gene Expression

#### 2.4.1. RPE Collection

RPE samples were collected from additional animals exposed to the following conditions: 1 day or 1 week of CL wear, as well as 1 day or 2 weeks of recovery from CL wear (Figure S1). To exclude the possibility of ocular developmental abnormalities, the animals were screened prior to the initiation of treatment at 2 weeks of age; both SERs and ALs were measured and any guinea pigs with interocular differences of more than 2.0 D in SER or 0.1 mm in AL were excluded. Only one animal met these criteria and was thus excluded.

The protocols used for RPE collection and RNA extraction from the RPE samples were as previously described [27]. Briefly, the guinea pigs were euthanized with 0.5 mL Euthasol (Virbac Animal Health, Ft. Worth, TX, USA) and then, their eyes were quickly enucleated and immediately immersed in chilled phosphate-buffered saline (PBS) buffer, after which scissors were used to open the eyes just behind the limbus, and anterior segments and retinas were carefully removed to isolate the posterior eye cups. The latter were then immersed in RNAlater stabilization solution (Invitrogen) for 5 min, after which a 1 mL syringe filled with PBS and a 30 g needle attached was used to gently detach the RPE from the choroid. The detached RPE cells were then collected in 1.5 mL tubes, spun down, and lysed with RLT buffer from RNeasy mini kits (Qiagen, Valencia, CA, USA). Total RNA was purified from the RPE samples using RNeasy mini kits (Qiagen), with on-column DNase digestion, according to the manufacturer’s protocol.

#### 2.4.2. Selection of RPE Genes for Analysis

**Figure 1:**
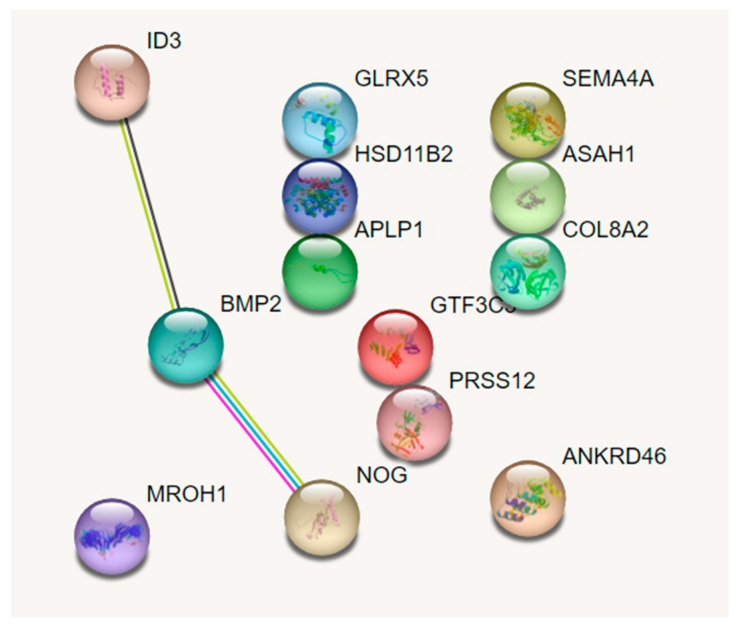
Protein–protein interaction analysis of differentially expressed genes in retinal pigment epithelium (RPE) after 1 day of monocular wear of a myopia-inducing contact lens (treated vs. fellow control eyes). The 13 genes addressed here were identified in the previous study involving RNA sequencing with the RPE. [23] Bone morphogenetic protein 2 (BMP2), inhibitor of DNA binding 3 (ID3), and Noggin (NOG) were identified as the interactive genes as determined using STRING (Search Tool for the Retrieval of Interacting Genes/Proteins), with the different colored lines distinguishing between the various types of evidence for association.

In our earlier, already published RNA sequencing study involving young guinea pigs, significant treatment-related interocular differences were observed in the expression of 13 genes in RPE collected after 1 day of exposure to a myopia-induced (−10 D CL) [23]. Using this result, protein–protein interaction analysis of differentially expressed genes was performed using the Search Tool for the Retrieval of Interacting Genes (STRING) database (Version 11.5: available at https://string-db.org/, accessed on 3 February 2023) [28]. Homo sapiens was selected as the organism. BMP2, ID3, and NOG were identified as interacting genes (Figure 1) and thus selected for further investigation.

#### 2.4.3. Quantitative Real-Time PCR (qRT-PCR)

The RNA samples were first reverse transcribed to cDNA (SuperScript III First-Strand Synthesis System for RT-PCR, Invitrogen, Carlsbad, CA, USA). Quanti-Tect SYBR Green PCR Kits (Qiagen) were used for mRNA amplification, along with a StepOnePlus Real-Time PCR System (Applied Biosystems, Foster City, CA, USA). Melt curves were examined to verify the yield of single-peak products. All real-time PCR reactions were performed in triplicate. The primer information used in the current study is summarized in Supplemental Table S1.

### 2.5. Statistical Analyses

All data are presented as the means ± SD. All data were tested for normality of distribution and equality of variance before the application of parametric statistics, with nonparametric tests applied to data not meeting these conditions. To allow for inter-animal differences between normal growth rates and responses to treatment, interocular differences (i.e., treated eye—fellow control eye) were used to express treatment effects. Repeated measures analyses of variance (ANOVA) were used to compare changes over time in interocular differences in SERs, ALs, ChTs, and ScTs. For gene expression data, the Wilcoxon signed-rank test was used to assess differences between treated and control eyes. *p*-values less than 0.05 were considered to be statistically significant. Statistical analyses were performed with JMP Pro version 14.3.0 (SAS Institute Inc., Cary, NC, USA).

## 3. Results

### 3.1. Changes in Biometric Parameters during Myopia Induction and Recovery

**Figure 2:**
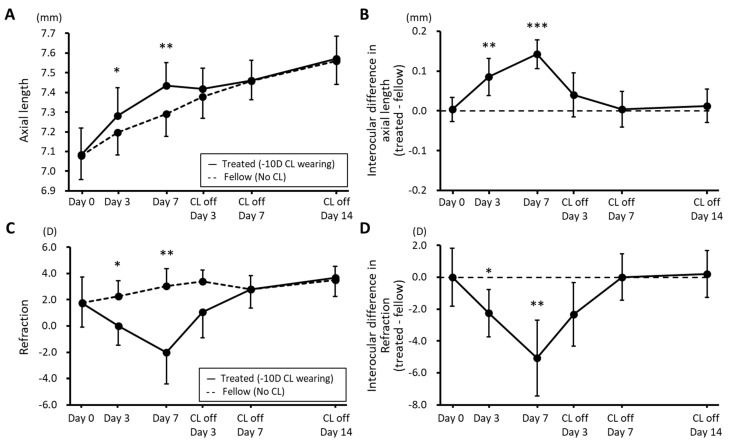
Axial lengths (**A**) and refractive errors (**C**) plotted against time for both eyes of guinea pigs wearing a −10 diopter contact lens (CL) on their right eye for 7 days, after which the lenses were removed and both eyes were tracked for a further 14 days. Also shown are interocular differences across the same 3-week experimental period for axial lengths (**B**) and refractive errors (**D**). (mean ± SD, n = 8; * *p* < 0.05; ** *p* < 0.01; *** *p* < 0.001).

*Axial Length and Refractive Error:* Figure 2 shows the time course of changes in ALs and SERs (n = 8). Compared to the ALs of the fellow (control) eyes, the ALs of the CL-wearing eyes had significantly increased after just 3 days (7.28 ± 0.14 vs. 7.19 ± 0.11 mm) and increased further by day 7 (7.43 ± 0.11 vs. 7.29 ± 0.12 mm) (*p* = 0.036 and 0.007, Figure 2A). However, these interocular differences rapidly disappeared after discontinuation of CL wear, with there being minimal interocular difference in ALs by day 7 of the recovery period (Figure 2A). Mean interocular differences (treated—control) in ALs were significantly different from baseline, after both 3 and 7 days of CL wear (0.00 ± 0.03 vs. 0.09 ± 0.05 and 0.14 ± 0.04 mm, resp.; *p* < 0.0001; Figure 2B). The same trends are evident in the SER data (Figure 2C,D).

**Figure 3:**
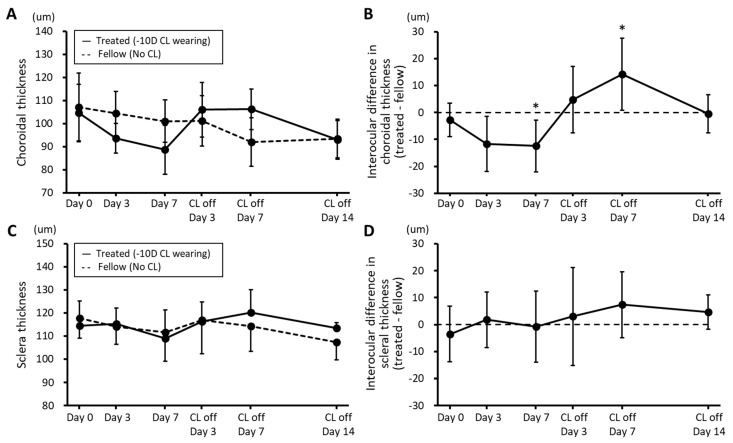
Choroidal thickness (**A**) and scleral thickness (**C**) plotted against time for both eyes of guinea pigs wearing a −10 D contact lens (CL) on their right eye for 7 days, after which the lenses were removed and the eyes were tracked for a further 14 days. Also shown are interocular differences across the same 3-week experimental period for choroidal thickness (**B**) and scleral thickness (**D**) (mean ± SD, n = 8; * *p* < 0.05).

*Changes in Choroidal and Scleral Thickness:* The choroids of eyes fitted with CLs showed significant thinning after 7 days of wear (*p* = 0.004, Figure 3A,B and S2). In contrast, after CL wear was discontinued, the previously observed ChT thinning relative to the fellow control eyes, i.e., during the induction phase, was replaced by sustained thickening, as reflected in the mean interocular difference in ChTs recorded on day 7 of the recovery period (14.2 ± 13.3 μm; *p* = 0.009; Figure 3A). This finding contrasts with the more rapid normalization of ALs, with treated eyes fully recovering within 7 days of CL removal. Nonetheless, ChTs had normalized by day 14 of the recovery period. Note that the ChT changes are approximately an order of magnitude smaller than the observed AL changes, although they would have contributed to the observed AL changes, i.e., increased elongation during CL wear and slowed elongation after the termination of wear. No significant interocular differences in ScTs were recorded over either the CL wearing or recovery periods (Figure 3C,D).

### 3.2. RPE Bmp2, Id3, and Nog Expression Changes with Myopia Induction and Recovery

#### 3.2.1. Normal Gene Expression

**Figure 4:**
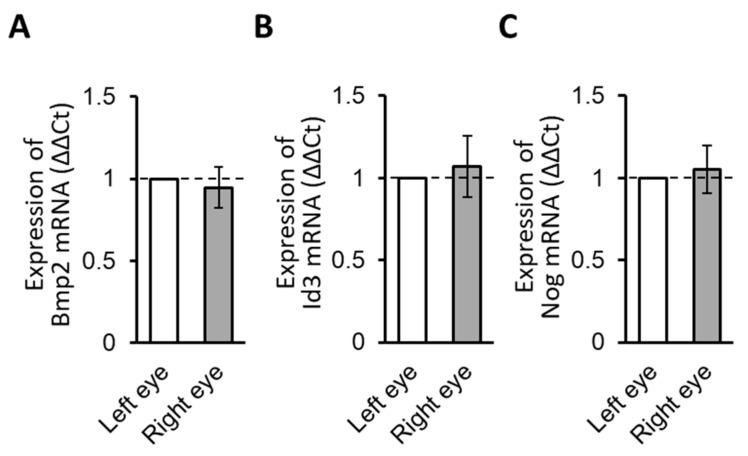
Bmp2 (**A**), Id3 (**B**), and Nog (**C**) gene expression, normalized to GAPDH, in RPE from the right and left eyes of untreated 15-day-old animals. The results shown represent relative mRNA expression (ΔΔ Ct for the fellow control eye = 1); no significant interocular differences between the left and right eyes were detected (n = 4).

RPE samples from the untreated animals were first analyzed with respect to *Bmp2, Id3, and Nog* gene expression levels to verify detectable expression levels and to examine normal interocular variations. As normalized to *Gapdh*, expression levels for all three genes in the RPE samples collected from untreated 15-day-old guinea pigs were measurable and the results for the right and left eyes were not significantly different (*p =* 0.64, 0.71, and 0.74; n = 4, Figure 4A–C, respectively).

#### 3.2.2. Myopia Induction

**Figure 5:**
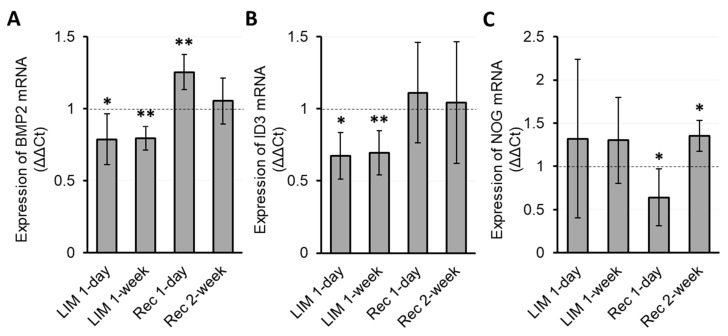
mRNA expression levels in the RPE of treated eyes compared to untreated fellow eyes for Bmp2, Id3, and Nog, normalized to GAPDH, after 1 day and 1 week of myopia induction (LIM), and 1 day and 2 weeks of recovery (REC), i.e., after removal of the myopia-inducing CLs. The plots show relative expression in the treated eyes compared to fellow control eyes for Bmp2 (**A**), Id3 (**B**), and Nog (**C**) (ΔΔ Ct for the fellow control eye = 1; unfilled bars). * *p* < 0.05 and ** *p* < 0.01.

The myopia-inducing effect of CL wear was coupled to downregulation in the expression of both *Bmp2* and *Id3* genes in RPE relative to expression levels in the RPE of contralateral eyes, detectable after just one day of CL wear, as well as on day 7, the last day of the CL wearing period, with changes for these two timepoints being similar in magnitude. Thus, after 1 day of CL wear, downregulation reached 78.7 ± 17.7 and 67.3 ± 16.1% for *Bmp2* and *Id3* genes, respectively (*p =* 0.012 and 0.016, resp.; n = 4, Figure 5A,B), while the equivalent values for 1 week of CL wear are 79.5 ± 8.3 and 69.6 ± 15.4% (*p =* 0.002 and 0.005, resp.; n = 6). On the other hand, no significant differential gene expression of Nog was detected after either 1 or 7 days of CL wear (*p* = 0.21 and 0.20, respectively; Figure 5C).

*Recovery from Induced Myopia:* After just 1 day of recovery, i.e., after CL wear had been discontinued for 1 day, *Bmp2* gene expression was upregulated (125.5 ± 12.1%, *p =* 0.004; n = 6, Figure 5A), while *Nog* gene expression was downregulated (64.1 ± 32.8%, *p =* 0.044, Figure 5C). The changes in Bmp2 gene expression were relatively short-lived, such that there was no significant difference in *Bmp2* expression between treated and fellow control eyes at 2 weeks into the recovery period and, likewise, no interocular difference *for Id3* gene expression. On the other hand, *Nog* gene expression in the “recovering eyes” was significantly increased in comparison with that of the fellow control eyes at the same 2 week timepoint, reversing the trend recorded earlier in the recovery period (135.6 ± 18.0%, *p =* 0.029; n = 4, Figure 5C).

### 3.3. Temporal Relationship between Gene Expression and Biometric Changes

Table 1 provides a schematic summary of the direction and magnitude of the changes described above. Overall, there appears to be a tight, albeit inverse relationship between the directions of AL and BMP2 gene expression changes, with ID3 gene expression changes mirroring BMP2 gene expression changes during myopia induction but not during recovery from the same treatment. Intriguingly, significant NOG gene expression changes were detected only during the recovery period, over which there was also a directional change from early downregulation, opposite to that recorded for BMP2 at the same time, to upregulation at the last 2-week timepoint, even while the ALs had normalized.

## 4. Discussion

This study made use of negative power (−10 D), rigid contact lenses to induce myopia in young guinea pigs, as previously described by our group. Consistent with other approaches making use of equivalent spectacle lenses [29] and form-deprivation protocols, the treated eyes showed accelerated elongation, leading to significant interocular differences in ALs after just 3 days, coupled with significant myopic changes in refractive error. In our earlier study, ALs were also measured after 1 day of CL wear, when no significant interocular difference was detected, although a significant difference was recorded after 5 days of wear [23].

The ability of young eyes to recover from induced myopia has been reported in a number of species, including chicks [30], guinea pigs [31], tree shrews [32], and rhesus monkeys [33]. In the study described here, we were able to confirm for our RGP-CL model that the eyes of young guinea pigs are able to recover from CL-induced myopia, at least when lens wear is terminated at a sufficiently early timepoint. Thus, when CL wear was terminated after 1 week of myopia induction, elongation was transiently halted in previously treated eyes, with induced myopia rapidly regressing and the eyes fully recovering in terms of both refractions and axial lengths after 1 week of recovery. The above patterns of myopia induction and recovery lend validity to this negative RGP-CL model, as an alternative to traditional spectacle lens models, for inducing myopia in young guinea pigs.

The important contribution of choroidal thickness changes to both induced myopia and recovery from the same treatment was confirmed for this CL model. A previous study using −4 D spectacle lenses to induce myopia in young guinea pigs reported a similar pattern of choroidal thickness changes during the development of and recovery from induced myopia, as observed using OCT imaging [34]. Specifically, 1 week of lens wear led to reduced ChTs and longer ALs, while 4 days after lens removal, ChT had significantly increased. That the CL-wearing eyes underwent choroidal thinning relative to their fellows in the study reported here is consistent with the latter findings, as is the observation of choroidal thickening during the recovery period, which together with the slowed elongation of the previously treated eyes accounted for the complete regression of induced myopia after just one week without CLs. Overall, the ChT changes were more sustained than the AL changes over the recovery period, although the ChT values had also normalized after 2 weeks of recovery.

Scleral thickness data were also extracted from in vivo biometric measurements. While myopia is typically linked to scleral thinning, no significant interocular difference in ScT was observed after 1 week of myopia induction. Likely contributing factors are the relatively small magnitude of induced myopia and the relatively short duration of treatment, although the scale of the changes may have also been below the resolution of the in vivo SD-OCT imaging system used in our study. While scleral ultrastructure was not examined in this study, myopia-related decreases in the scleral collagen fiber cross-sectional areas were observed in a previous electron microscopy study involving guinea pigs subjected to longer term myopia induction treatments, either 6 weeks of negative lens wear or 10 weeks of form deprivation [35,36]. The latter findings also have their parallel in observations of scleral thinning from highly myopic human eyes [37,38]. Follow-up studies to investigate the time frame for the development of such myopia-related scleral changes and their potential reversibility are warranted.

As summarized in the introduction, there is now general consensus that early ocular growth regulation is mediated by local circuits within the eye, originating in the retina. In the case of the defocus model used in the current study, evidence suggests that the retina can decode the direction of the imposed or induced optical defocus, i.e., hyperopic, as in the case of the negative CL used here, or myopic defocus, as experienced when the CL was removed, to generate a signal that ultimately modulates the thickness and/or growth of the choroidal and scleral walls of the eye. In this ocular growth regulation model, the RPE is assumed to play a key role as a signal relay linking the retina and these outer ocular tissues, with the gene expression data reported here and earlier findings from chicks [24,25,39] providing supporting evidence. Indeed, the latter provided some of the motivation for this study.

To date, most relevant gene expression studies, including more recent RNA-Seq analyses, have targeted either the ocular choroid or the combined retina–RPE complex. Examples of the former include studies in guinea pigs, tree shrews, and marmosets [15,40,41]. Studies focusing on the RPE have been largely limited to the chick model, with a consistent finding across a series of studies undertaken by Zhang et al. being the early, bidirectional regulation of Bmp2, which was found to be rapidly downregulated with the initiation of myopia-inducing treatments (both form deprivation and imposed hyperopic defocus) and rapidly upregulated under conditions that slow eye elongation, such as those created by the termination of myopia-inducing treatments [24,25,39]. The results of the present study in guinea pigs are consistent with results from these studies in chicks, specifically, Bmp2 gene expression was downregulated in the RPE after the initiation of our myopia-inducing CL treatment but upregulated when this treatment was terminated to reveal uncorrected, induced myopia. Specifically, whereas Bmp2 gene expression was significantly decreased in the RPE of the treated eyes, 1 day and 1 week after the initiation of CL wear, Bmp2 gene expression was significantly increased 1 day after CL removal, returning to normal 2 weeks after CL removal. That the expression of Bmp2 remained downregulated over the CL wearing period is consistent with the continued accelerated elongation of the treated eyes, which, in refractive terms, had not yet fully compensated for the imposed hyperopic defocus. On the other hand, while both refractive errors and eye lengths had normalized by the end of the 2-week recovery period, along with Bmp2 gene expression, the choroids of previously treated eyes remained thickened over the same recovery period. While the close correspondence in the findings for RPE-Bmp2 gene expression in chicks and guinea pigs suggests a key role in defocus-modulated eye growth, the latter discrepancy also points to a more complex picture, as it relates to the regulation of choroidal thickness.

In this study, we also examined treatment-related RPE expression changes in two additional genes, Id3 and Nog genes. These two genes were identified in protein–protein interaction analysis targeting Bmp2, which was 1 of 13 genes whose expression was found to have changed significantly after one day of CL wear in our previous RNA sequencing study involving guinea pig RPE. With respect to the functional relationships between these three genes, Id3 is a transcription factor downstream of BMP signaling while Nog is an inhibitor of BMP [42,43,44]. As reported in our previous RNA sequencing study, Id3 gene expression was found to be downregulated across the CL wearing (myopia-inducing) period, i.e., after both one day and one week of CL wear, and consistent with the pattern of downregulation for Bmp2. On the other hand, during the “myopia recovery” phase, changes in Id3 expression did not reach statistical significance, despite a trend towards an increase. No clear trends are evident in the expression data for the Nog gene corresponding to the myopia induction phase, although a significant, presumed transient decrease one day after CL removal was observed, followed by an increase two weeks into the recovery period. Together, these findings strongly implicate all three genes in the proposed molecular signaling pathway mediating defocus-driven eye growth, providing further indirect evidence for a retinal defocusing decoding mechanism with capacity to encode the direction (i.e., sign) of the imposed defocus. The more complex pattern of expression changes for the Nog gene is also consistent with a complex signal relay involving feedback regulation.

In this study, we examined three gene expression changes in Bmp2, Id3, and Nog in RPE during myopia induction and subsequent recovery from myopia. It represents the first study to examine RPE expression changes for these genes in the context of myopia induction and recovery in any mammal or primate. The results reported here not only add to the evidence from our earlier guinea pig study linking the downregulation of Bmp2 to myopia progression, but, importantly, they add to the evidence from our previous studies in chicks [25,39] for its bidirectional regulation, with imposed myopic defocus, as experienced during the recovery period, upregulating Bmp2 gene expression.

Can these documented gene expression changes be translated into a therapy for controlling myopia progression? The influences of Bmp2, albeit complex, on the early development of various ocular tissues is now well recognized [45,46,47]. While retinal dopamine has been linked to eye growth regulation, with reduced turnover linked to accelerated eye growth in a number of animal models [17,48,49], whether there is a direct link between retinal dopamine and RPE BMP2 gene expression changes is yet to be investigated. Other possible follow-up studies include the evaluation of changes in Bmp2 protein levels in the choroid under the conditions shown to yield gene expression changes in the current study [50,51]. Beyond the current study, the possibility that differences in choroidal thickness observed between the two strains of pigmented guinea pigs may be linked to differential gene expression within the RPE warrants investigation [52]. It may also be insightful to investigate whether reported differences between form-deprivation myopia and lens-induced myopia extend to the RPE gene expression level [53]. While overall, the accumulating evidence points to BMP2 serving as a negative growth regulator, further investigations are required to understand its exact roles in the choroidal and scleral changes underlying changes in eye size and thus refractive error [54].

Guinea pigs and humans demonstrate intriguing contrasts and convergences in the context of vision, with retinal organization and processing being of greatest relevance to the study at hand. Unlike traditional nocturnal rodent models, guinea pigs are crepuscular, being visually active during daylight hours, just as humans [55]. For the same reason, their visual acuity is superior to their close rodent relatives but, nonetheless, inferior to human visual acuity, as guinea pig retina lacks a fovea [56]. Despite this constraint, other characteristics of the guinea pig’s visual system, including their cone-rich retina, is reflected in their popularity as a model for investigating various aspects of visual processing [57,58]. In attempting to translate myopia-related eye growth regulation studies from guinea pig to humans, these same studies may offer important insights into the strengths and limitations of this guinea pig model.

In summary, the study reported here has further validated this rigid CL model of myopia induction as an alternative to traditional spectacle lens models, at least when applied to young guinea pigs, while also providing further confirmatory evidence that the eyes of young animals can recover from induced myopia, with the observed strong inhibitory effect of axial elongation along with choroidal thickening arguing for an underlying active regulatory process. The observed bidirectional changes in RPE Bmp2 gene expression, closely tied to ocular biometric changes, i.e., in AL and ChT, provide confirmatory evidence for results from earlier studies in chicks and warrant further studies into underlying molecular mechanisms and their potential therapeutic application for myopia progression control.

## Figures and Tables

**Table 1:** The relationship between changes in ocular biometrics and gene expression in the RPE.

	Measurement Timepoints				
Parameters	1–3 Days LIM	1 Week LIM	1–3 Days Rec	1 Week Rec	2 Weeks Rec
Axial length	**↑**	**↑↑**	**↓**	**→**	**→**
Choroidal thickness	**→**	**↓**	**↑**	**↑↑**	**→**
BMP2 expression	**↓**	**↓**	**↑**	N/A	**→**
ID3 expression	**↓**	**↓**	**→**	N/A	**→**
NOG expression	**→**	**→**	**↓**	N/A	**↑**

**↑** or **↓**: significant increase or decrease (*p* < 0.05), **↑↑**: significant increase (*p* < 0.01), →: no difference compared to the control eye, LIM: lens induced-myopia, Rec: recovery, RPE: retinal pigment epithelium, N/A: not available.

## Data Availability

The data are available upon reasonable request.

## Supplementary Materials

The following supporting information can be downloaded at: https://www.mdpi.com/article/10.3390/biom13091373/s1. Table S1: Nucleotide sequences of primers used for PCR amplification; Figure S1: Study design, showing the contact lens (−10 D) wearing schedule, along with the timelines for in vivo measurements and the collection of retinal pigment epithelium (RPE) samples for mRNA isolation. The black arrowheads indicate the time points at which refractive error, axial length, as well as choroidal and scleral thickness data were collected; the red vertical bars indicate the time points at which the RPE samples were collected. LIM: lens-induced myopia. Figure S2: Representative SD-OCT fundus images from treated and fellow eyes of the same guinea pig captured 1 mm above the optic nerve head, on day 0 (pretreatment baseline), after 1 week of CL wear (−10 diopter), and week 1 and week 2 after termination of CL wear. Retinal, choroidal, and scleral boundaries are clearly visible in all images.
