# Supplementary material for: Changes in Expression in BMP2 and Two Closely Related Genes in Guinea Pig Retinal Pigment Epithelium during Induction and Recovery from Myopia
Source: Biomolecules. 2023 Sep 11;13(9):1373. doi: 10.3390/biom13091373 (PMC10526436; doi:10.3390/biom13091373)
Supplement: Supplementary file 1 [file biomolecules-13-01373-s001.zip › biomolecules-2506581-supplementary.pdf]

Table S1. Nucleotide sequences of primers used for PCR amplification

Gene	Forward Primer (5' to 3')	Reverse Primer (5' to 3')
Bmp2	ATCCTGAGCGAATTCGAGTTG	GGGTGGGTCTCTGCTTCAAG
Id3	AACAGAGCCTTTCTCCAAGGAA	TGTCCGAACTCTGCCAAGGT
Nog	GGTCGAAGATAGGGTCCAAGTG	TCCAATTCCCAGCGACAAC
Gapdh	GCAACCCGAGACAAGATGGT	GCGTCCAATACGG CCAAAT

Bmp2, bone morphogenetic protein 2; Id3, inhibitor of DNA binding 3; Nog, noggin; Gapdh, glyceraldehyde-3-phosphate dehydrogenase.

Supplementary Materials Figures

Figure S1

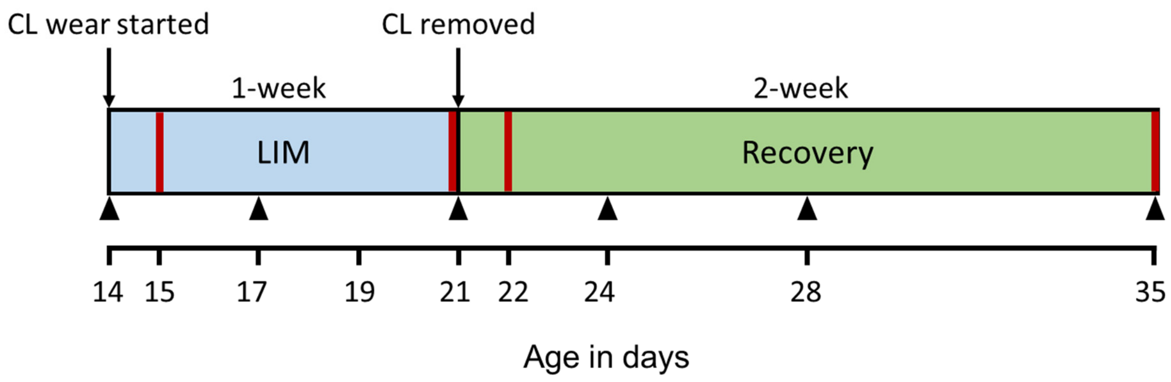

Figure S1**.** Study design, showing the contact lens (-10 D) wearing schedule, along with the time lines for *in vivo* measurements and collection of retinal pigment epithelium (RPE) samples for mRNA isolation. The black arrowheads indicate the time points at which refractive error, axial length, as well as choroidal, and scleral thickness data were collected; the red vertical bars indicate the time points at which RPE samples were collected. LIM: lens-induced myopia.

Supplementary Materials Figures

Figure S2

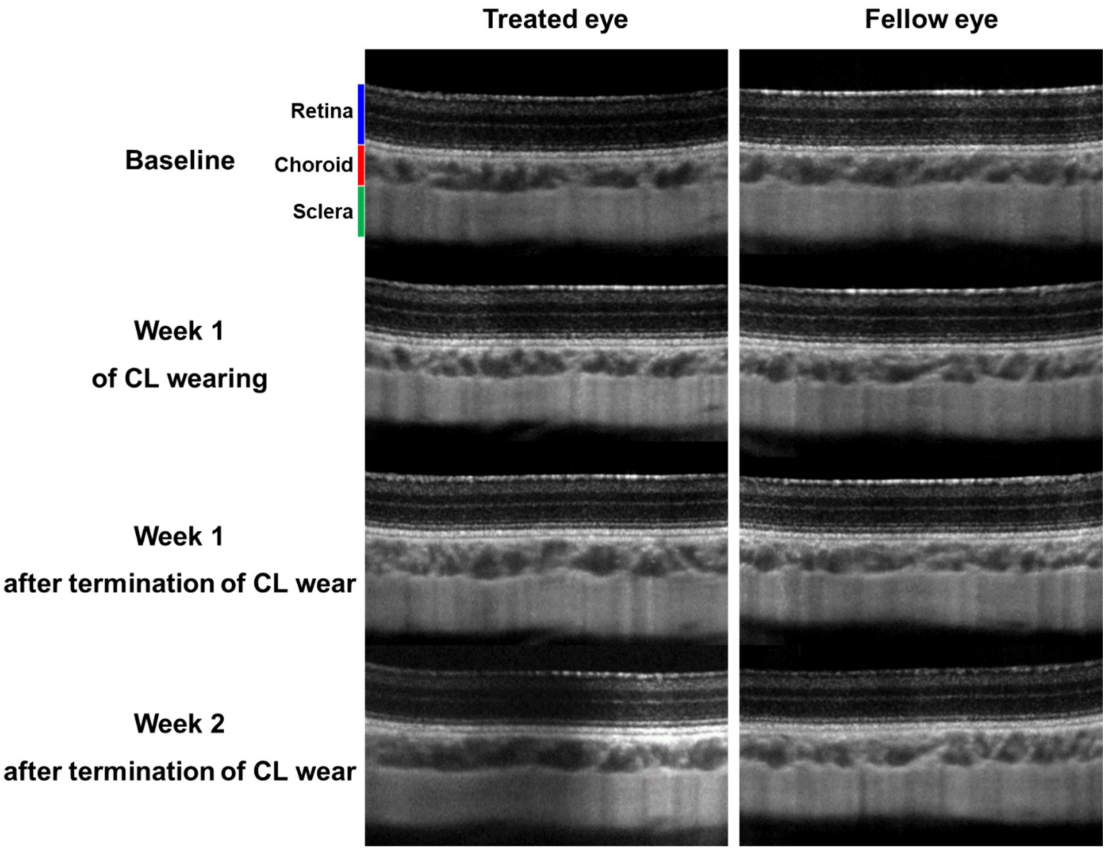

Figure S2: Representative SD-OCT fundus images from treated and fellow eyes of the same guinea pig captured 1 mm above the optic nerve head, on day 0 (pretreatment baseline), after 1 week of CL wear (-10 diopter), and week 1 and week 2 after termination of CL wear. Retinal, choroidal, and scleral boundaries are clearly visible in all images.
